# Ultra‐Low Operating Voltage Memristors Based on Plating/Stripping Reactions

**DOI:** 10.1002/advs.202510370

**Published:** 2025-07-21

**Authors:** Lingbo Yao, Zhurui Wang, Yanyu Sun, Xiaowei Chi, Yu Liu

**Affiliations:** ^1^ Shanghai Institute of Ceramics Chinese Academy of Sciences Shanghai 200050 China; ^2^ University of Chinese Academy of Sciences Beijing 100049 China

**Keywords:** Butler–Volmer equation, deep eutectic gel electrolyte, memristor, plating/stripping reaction

## Abstract

Current memristor technologies remain limited by instability, high operating voltage, and low switching ratio, primarily due to stochastic filament formation and defect migration. Here, a fundamentally different electrochemical mechanism is proposed through the development of a plating/stripping memristor (PSM) featuring stable, low‐voltage, and bio‐inspired conductance switching. Constructed with Zn/Cu electrodes and a deep eutectic gel electrolyte (DEGE), the PSM accurately emulates spike‐rate‐dependent plasticity and long‐term synaptic dynamics. The DEGE matrix offers a corrosion‐resistant, dendrite‐free, and ionically homogeneous environment, facilitating gradual and programmable conductance evolution. Remarkably, the Zn/DEGE/Cu PSM exhibits switching behavior with a low‐resistance state centered at 15.3 µV and dual high‐resistance states at –10.0 mV and +11.1 mV, governed by electrochemical equilibrium, highlighting its sub‐millivolt‐level operation and energy‐efficient switching characteristics. Furthermore, the Zn/DEGE/Cu PSMs are integrated into a reservoir computing framework using 4‐bit pulse‐encoded conductance states. When applied to pattern recognition tasks, the DEGE‐based PSM system demonstrates a reliable classification accuracy of 89.3%, driven by device‐derived temporal dynamics. Overall, this study establishes a new materials and mechanistic foundation for energy‐efficient neuromorphic computing, bridging electrochemical reactions with biologically plausible information processing.

## Graphical abstract

A plating/stripping memristor exhibits mV‐level redox switching, high endurance, and ultralow‐voltage operation. The system supports gradual conductance evolution and enables 4‐bit pulse‐coded reservoir computing, offering a physically interpretable platform for neuromorphic processing based on interfacial electrochemical kinetics.

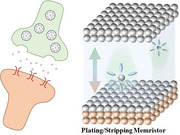

## 1 Introduction

The memristor, recognized as the fourth fundamental circuit element following resistors, inductors, and capacitors, has been extensively studied for storage and computing applications.^[^ ^1^, ^2^ ^]^ Traditional memristors primarily rely on oxide‐based mechanisms, such as oxygen vacancy migration in materials like TiO₂ and HfO₂, or ion‐exchange‐based E‐RRAM technology.^[^ ^3^ ^]^ These drawbacks are often rooted in the stochastic nature of conductive filament formation, oxygen vacancy drift, and interfacial degradation under repetitive cycling.^[^ ^4^, ^5^, ^6^ ^]^ What's more, these systems face several challenges including fabrication complexity, poor scalability, and inconsistent switching behavior.^[^ ^7^, ^8^ ^]^ Therefore, the development of memristors with enhanced reliability, energy efficiency, and ease of fabrication is crucial to advancing their practical application.^[^ ^3^, ^9^, ^10^, ^11^ ^]^

These challenges have motivated the development of alternative memristive systems capable of achieving low‐power operation, high‐speed switching, and improved endurance.^[^ ^12^, ^13^, ^14^ ^]^ Recent attention has turned to electrochemical systems, particularly those involving metal ion plating and stripping processes, which are well‐established in energy storage technologies.^[^ ^15^, ^16^ ^]^ Compared to defect‐mediated mechanisms, such electrochemical processes offer greater reversibility, interface‐localized control, and predictable reaction kinetics.^[^ ^17^ ^]^ Memristors based on metal ion migration and interfacial redox reactions exhibit several distinctive advantages: their switching dynamics can be quantitatively described using electrochemical models, conductance states can be modulated gradually and programmably through ion electromigration, and the architecture is compatible with a wide variety of solid, liquid, or gel electrolytes.^[^ ^18^, ^19^, ^20^ ^]^ Moreover, these systems can naturally emulate the analog and hysteretic characteristics of biological synapses, rendering them particularly well‐suited for neuromorphic and bio‐integrated electronics.

In this study, a novel memristive switching approach based on plating/stripping reactions is presented within a simple Zn/Cu electrochemical system. The core mechanism of the plating/stripping memristor is governed by interfacial redox kinetics in accordance with the Butler–Volmer model, distinguishing itself from conventional oxide‐based memristors. This new strategy enables stable and controllable memristive behavior with a high resistance ratio exceeding 10^5^ at sub‐0.1 V switching operation and non‐volatile state retention beyond 10^3^ s. Furthermore, by integrating the PSM array into a reservoir computing framework with 4‐bit pulse‐encoded input states, device‐intrinsic dynamics are directly exploited for temporal information processing. The system successfully performs handwritten digit recognition with conductance‐based state evolution, underscoring its potential for analog neural feature extraction. This work not only demonstrates a promising low‐voltage, low‐power alternative to traditional memristor technologies, but also introduces a versatile platform for future applications in neuromorphic computing, soft electronics, and biocompatible memory systems.

## 2 Results and Discussion

### 2.1 The Basic Principles of Electrochemical PSM

**Figure 1:**
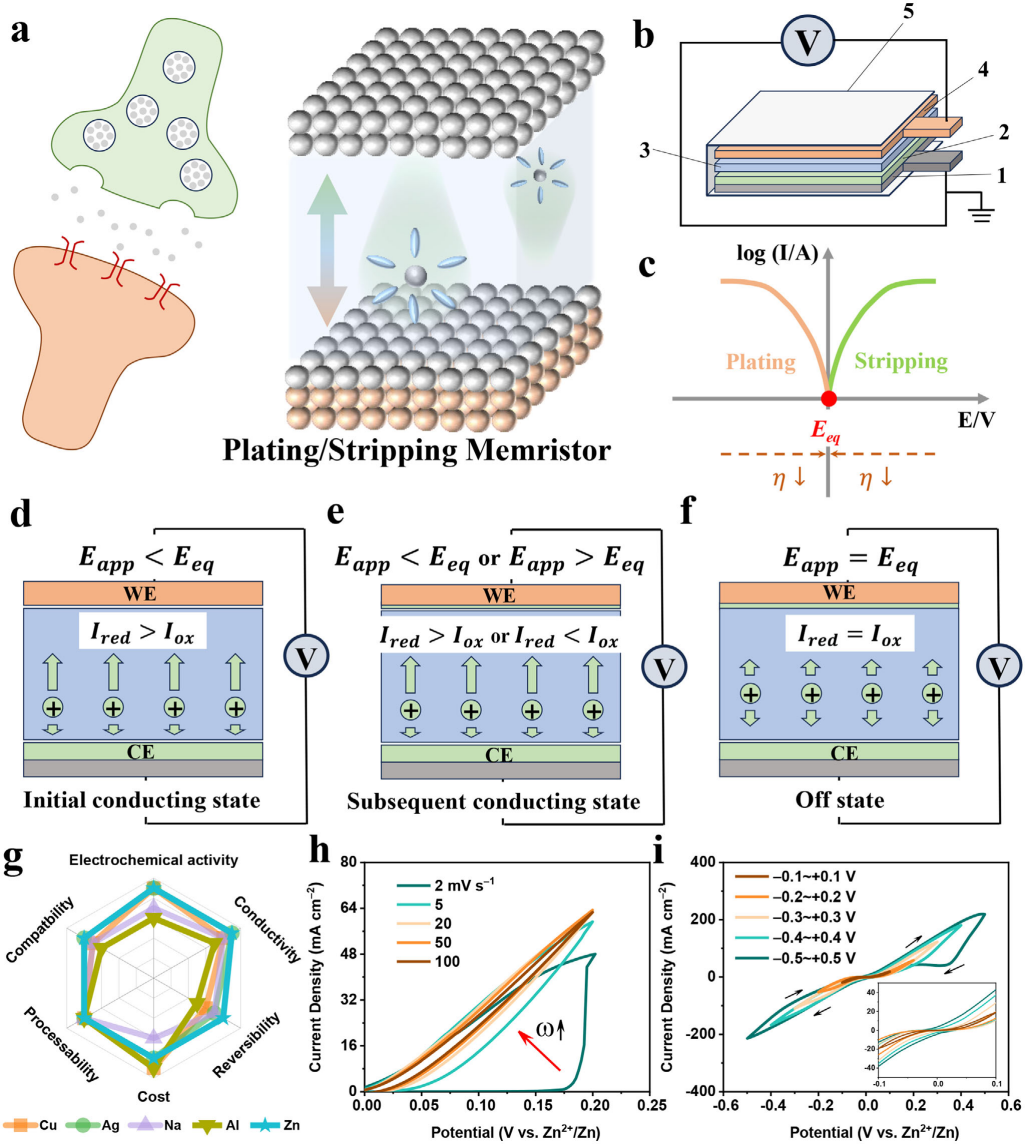
Basic electrochemical mechanism of the PSM memristor. a) Schematic illustration of the analogy between neuronal excitation/inhibition behaviors and electrochemical plating/stripping reactions. b) Schematic diagram of the structure of the PSM device (Component 1: the substrate electrodes, Component 2: the plating/stripping active metal, Component 3: the electrolyte material with ion‐conduction properties, Component 4: conductive substrate electrode, and Component 5: packaging layer). c) Typical Tafel ‘log I–V’ curve of the metal electrode with electrochemical activity. d) PSM in the initial conducting state when the reduction current *I_red_* on the working electrode (WE) exceeds the oxidation current *I_ox_* under *E_app_* < *E_eq_* (CE: Counter Electrode). e) PSM in the subsequent conducting (ON) state when the *I_red_* is not equal to the *I_ox_* under *E_app_* < *E_eq_* or *E_app_* > *E_eq_*. f) PSM in the insulating (OFF) state when *I_red_* =  *I_ox_* under *E_app_* = *E_eq_*. g) Comparative analysis of the overall performance of common plating/stripping active metal electrodes. The I–V curves of Zn/AE/Cu PSM under h) different scan rates from 2 to 100 mV s^−1^, and i) at different operating windows.

Unlike traditional oxide memristors, the PSM operates via the reversible plating and stripping reactions on active metal electrodes, without relying on oxygen vacancies or tunneling barriers. This mechanism provides higher stability, material versatility, and lower processing costs. As shown in **Figure** **1**, the core electrochemical behaviors and switching state transitions are illustrated. The electrochemical plating and stripping reactions not only modulate conductance but also bridge classical electrochemistry with biological learning processes. The plating and stripping reactions at the electrode interface mirror synaptic excitation and inhibition, respectively (Figure 1a). Both systems exhibit ion fluxes, directional and time‐dependent behavior, and history‐dependent plasticity. This intrinsic alignment with biological function offers a physics‐native implementation of synaptic behavior, contrasting with digital emulation or resistive switching mechanisms. The applied bias and interfacial kinetics create a chemical potential landscape that serves as the learning substrate, making the device function similarly to biological processes. The PSM features a typical encapsulated sandwich structure (Figure 1b), ensuring efficient redox reactions and mechanical stability, making it suitable for neuromorphic applications.

The log *I* − *V* characteristic diagram in Figure 1c reveals the similarity between the PSM and traditional memristor voltammetry characteristics. Both of them exhibit significant changes in current response due to potential variations and involve the dynamic evolution of charge transfer and mass transfer processes. However, the core mechanism can be derived from the classical Butler‐Volmer relation, as shown in Equation (1):^[^ ^21^ ^]^

(1) $$I=I_{0}\left[\exp\left(\frac{\alpha nF\eta }{RT}\right)-\exp\left(-\frac{\left(1-\alpha \right)nF\eta }{RT}\right)\right]$$

where *I* is the current density, *I*_0_ is the exchange current density for the plating/stripping reaction, α is the transfer coefficient, *n* is the number of electrons transferred, *F* is the Faraday constant, η is the overpotential, *R* is the gas constant, and *T* is the temperature. This conceptual innovation enables the reinterpretation of synaptic weight change as a dynamic redox process, where the extent of metal plating (potentiation) or stripping (depression) is finely tunable through the input stimuli (Figure 1d‐f). At voltages below the Nernst equilibrium (Applied Potential *E_app_* < Equilibrium Potential *E_eq_*), spontaneous Zn plating occurs, leading to a behavior mirroring long‐term potentiation (LTP) in biological synapses. Conversely, biasing the system above *E_eq_* promotes stripping, emulating long‐term depression (LTD). The ability to maintain the conductance state near equilibrium with ultra‐low energy input adds a non‐volatile resting behavior, akin to synaptic quiescence. It reflects the device's ability to integrate voltage‐time histories and store them as stable, analog conductance states. These emergent phenomena demonstrate that electrochemical dynamics, long studied for energy applications, can be repurposed as a computational primitive, offering a new material and mechanistic paradigm for neuromorphic systems.

Figure S1 (Supporting Information) compares representative redox couples and their standard electrode potentials that are thermodynamically favorable for reversible plating/stripping processes. Considering switching dynamics, material cost, and fabrication compatibility, transition and post‐transition metals such as Fe, Ni, Cu, Zn, Cd, Sn, and Pb—well‐suited to aqueous electrolyte environments—emerge as promising candidates. From a mechanical reliability perspective, metals with moderate Young's modulus and hardness are preferred, as they effectively suppress dendritic growth and mechanical failure during high‐frequency and long‐term operation (Figure S2, Supporting Information).^[^ ^16^ ^]^ Aqueous and hydrogel‐based electrolytes offer superior ionic conductivity, facile processing, and potential biocompatibility, making them particularly attractive for next‐generation flexible and bioelectronic platforms (Figure S3, Supporting Information). As shown in the radar chart (Figure 1g), Cu, Na, and Zn demonstrate optimal balances among electrochemical reversibility, conductivity, redox activity, and manufacturability. Among them, Zn and Na offer distinct advantages in cost‐effectiveness and large‐scale applicability, while Zn additionally provides higher coulombic efficiency and operational stability. Based on these considerations, a Zn‐based PSM (Zn/Aqueous Electrolytes/Cu configuration, simplified as Zn/AE/Cu) was selected for further investigation, exhibiting superior redox reversibility and stable conductance modulation over extended cycling compared to its Na‐ and Cu‐based counterparts (Figures S4 and S5, Supporting Information).

The variable scan rate I–V curves in Figure 1h demonstrate that the Zn/AE/Cu PSM system exhibits typical memristive behavior. When a *E_app_* (0 V vs. Zn^2+^/Zn) was applied, the Zn/AE/Cu PSM maintains a near‐zero current density, indicating that the conductance modulation is primarily governed by the Butler–Volmer relationship, proving the plating/stripping‐based memristive mechanism. As the scan rate increases, the I–V curves become more compressed, showing that at high scan rates, the Zn/AE/Cu PSM is limited by diffusion kinetics rather than charge transfer kinetics, which is a hallmark of memristors.^[^ ^22^ ^]^ Higher switching frequencies and R_OFF_/R_ON_ ratios contribute to improved response speed and overall performance. Notably, the Zn/AE/Cu PSM system exhibits a low switching voltage (< 0.5V), which is crucial for low‐power memristor applications. Figure 1i shows that even at an ultra‐low switching voltage of 0.1 V, the device still maintains highly reversible switching behavior (R_OFF_/R_ON_ = ≈10⁴, Figure S6, Supporting Information). Within the 0.4–0.5 V operating window, the R_OFF_/R_ON_ ratio exceeds 10⁵, surpassing the performance of many conventional memristors reported in the literature.

### 2.2 AE‐Based PSM: Performance and Challenges

**Figure 2:**
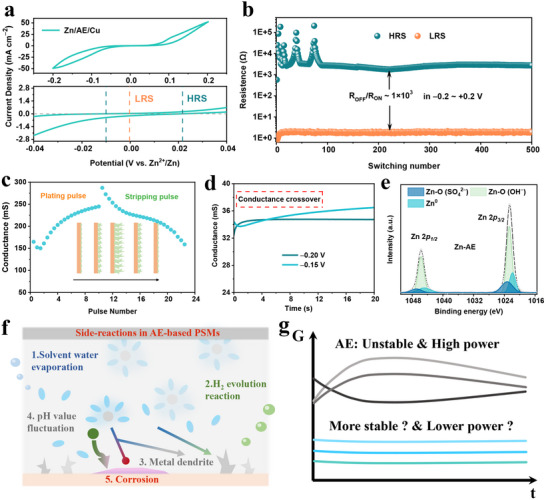
The memristive behaviors and interfacial issues of AE‐based PSM. a) The switching of the Zn/AE/Cu PSM. b) Endurance of LRS and HRS resistances with 500 continuous DC switching cycles. c) The conductance obtained from the stimulation with multiple pulses is used to simulate synaptic weight change. d) The conductance crossover phenomenon of AE‐based PSM at −0.15 and −0.20 V. e) The Zn 2*p* XPS spectra of the electrode interface using AE. f) Key issues at the electrode/electrolyte interface of AE‐based PSMs. g) Time‐dependent evolution of conductance of AE‐ and DEG‐based PSM devices.

**Figure** **2**a demonstrates the typical I‐V characteristics of the Zn/AE/Cu PSM, showing bipolar switching behavior with clear differentiation between low‐resistance state (LRS) and high‐resistance state (HRS). The transition between these states is dominated by the migration of Zn^2^⁺ ions, confirming the electrochemical switching mechanism driven by plating/stripping. In the HRS, the current density is near zero, while in the LRS, it increases significantly, indicating effective memristive switching. The ability to reliably switch between these states under voltage application is a key feature for non‐volatile memory in neuromorphic systems. Figure 2b shows the resistance switching behavior of Zn‐based over 500 cycles, with a high R_OFF_/R_ON_ ratio (≈10^3^) at an operating window of −0.2–+0.2 V, demonstrating more stable switching behavior than Cu‐based PSM (Figure S7, Supporting Information). As shown in Figure S8 (Supporting Information), the consistency in HRS/LRS with minimal fluctuation across different Zn‐based PSMs highlights the promising application potential of this approach. These characteristics underscore the PSM's potential for robust, high‐performance non‐volatile memory applications, showcasing its effectiveness in long‐term switching behavior and consistent data retention.

During the plating and stripping processes, Zn/AE/Cu PSMs exhibit LTP and LTD mechanisms, which are key to mimicking biological synaptic behavior and enabling future brain‐inspired computing technologies for storage and computation integration. Figure 2c shows that the conductance obtained from the stimulation with multiple pulses is used to simulate synaptic weight change characteristics (Figure S9, Supporting Information), where the evolution is associated with plating (LTP) and stripping (LTD), respectively. During plating (*E_app_*  <  *E_eq_*), Zn^2^⁺ migrate and deposit to form a high surface area activated zinc layer, enhancing the device's conductance, similar to the LTP in biological synapses. As the voltage switches to *E_app_* > *E_eq_*, the stripping process begins, where the Zn layer gradually dissolves under the positive pulse voltage, leading to a decrease in conductance, mimicking LTD behavior in biological synapses. Figure S10 (Supporting Information) shows the paired‐pulse facilitation (PPF) behavior of Zn/AE/Cu PSM, reflecting the device's short‐term plasticity characteristics. At shorter pulse intervals, the PPF index increases, indicating that the response to the second pulse is enhanced, similar to the LTP phenomenon in biological synapses, demonstrating the device's potential for mimicking neuromorphic learning and memory. However, dendrite growth introduces the risk of instability, as the formation of metal filaments may lead to device failure due to short circuits, demonstrating the delicate balance required for stable switching.

Figure 2d reveals that the AE‐based PSM exhibits severe conductance fluctuations (conductance crossover phenomenon) when subjected to different applied bias voltages, which is highly detrimental to the consistency and reliability of the memristive behavior. This instability in conductance switching indicates that the electrochemical process is not fully stabilized at certain voltage thresholds, resulting in inconsistent switching behavior over time. Figure 2e and Figure S11 (Supporting Information) present the XPS spectra of the used Cu electrode, showing significant corrosion and oxidation layers on the electrode surface, which are further exacerbated by the basic salts in the electrolyte. The quantified Zn^0^/Zn^2+^ ratio is only 0.26, indicating considerable oxidation and corrosion of the zinc layer during the operating process. These side reactions, such as hydrogen evolution and pH fluctuations, exacerbate the dissolution of the metal layer during stripping, further limiting the device's durability. Figure 2f illustrates these side reactions, including solvent evaporation, dendrite formation, and corrosion, which collectively undermine the long‐term stability and performance of the PSM. These findings highlight that controlling the plating and stripping processes, particularly managing dendrite growth and metal layer dissolution, is crucial for enhancing the stability and reliability of Zn/AE/Cu PSMs in neuromorphic applications.

Figure 2g highlights the challenges related to conductance stability and power consumption in the proposed PSM. As previously discussed, PSMs based on AE exhibit unstable conductance behavior and high power consumption, leading to poor reliability over long‐term use. Based on the derived conductance evolution during the plating and stripping processes, it is evident that electrolytes with large time constants and stable interfaces are necessary to achieve more stable conductance and low‐power characteristics. Deep eutectic gel electrolytes, with their low evaporation enthalpy, moderate interface dynamics, and high electrochemical stability, represent a promising solution to the challenge of constructing highly stable ionic conduction layers, thus ensuring enhanced reliability and stability for long‐term performance in PSM applications.^[^ ^23^, ^24^ ^]^

### 2.3 DEGE‐Based PSM: Performance and Prospect

**Figure 3:**
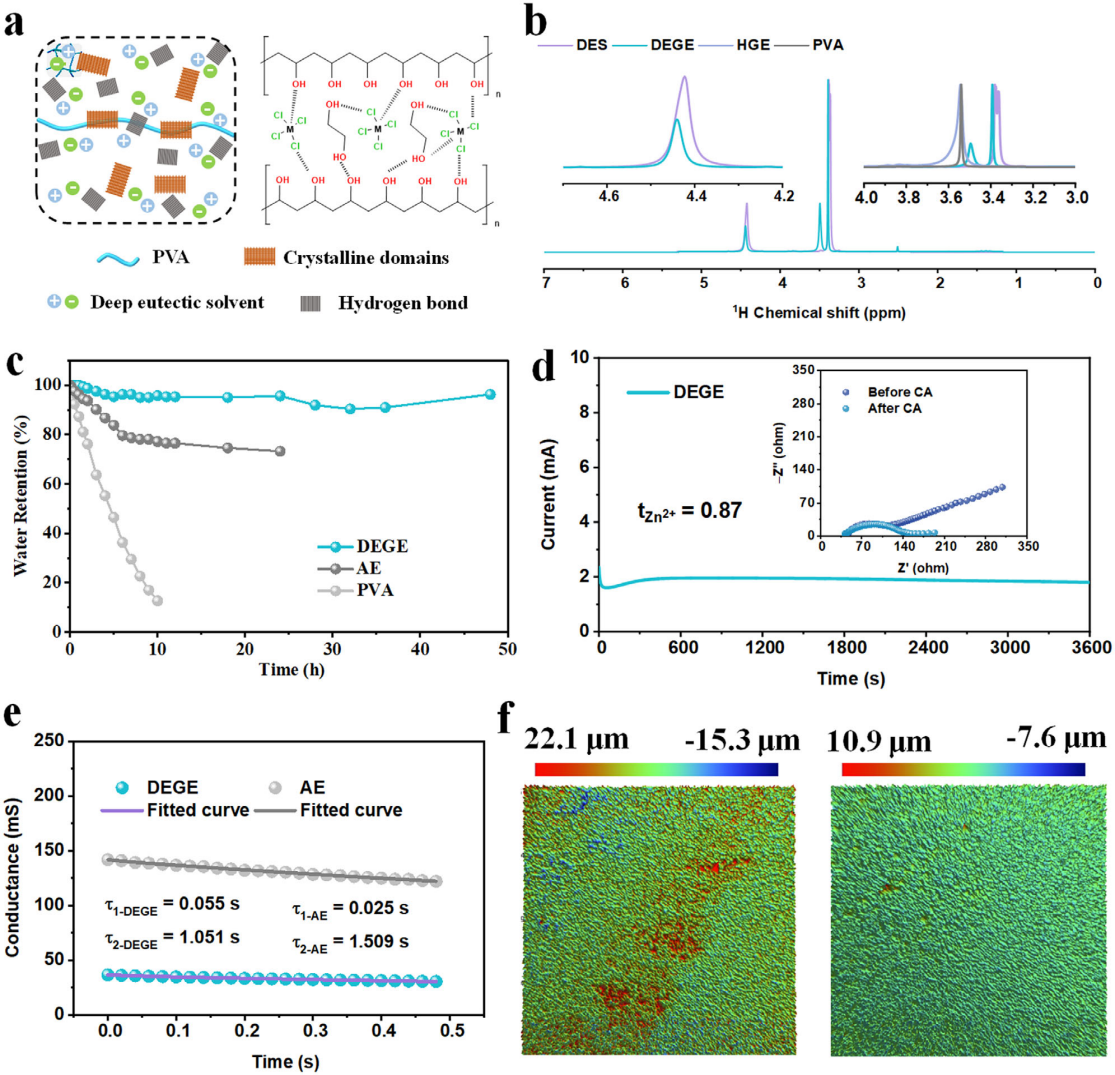
The design strategy and structural stabilization mechanism of DEGE. a) The illustration diagram of meso/micro structures in DEGE. b) The NMR ^1^H spectra of DES, DEGE, HGE, and PVA. c) The water retention tests of DEGE, AE, and PVA. d) The Zn^2+^ transference number of DEGE. e) The conductance evolution trends and model fitting of PSM for DEGE and AE under the pulse voltage of −0.005 V. f) The LSCM images of electrodes using AE and DEGE (right).

To address the volatility, side reactions, and instability associated with aqueous electrolytes, a deep eutectic gel electrolyte (DEGE) was designed by incorporating an ethylene glycol (EG)–ZnCl₂‐based deep eutectic solvent (DES) into a polyvinyl alcohol (PVA) matrix. As illustrated in **Figure** **3**a, the resulting hybrid structure forms an interconnected hydrogen‐bonding network among PVA hydroxyls, EG, and Zn–Cl coordination complexes, establishing a balanced ionic conduction framework between crystalline domains and amorphous channels. The FTIR spectrum of DEGE (Figure S12, Supporting Information) exhibits a broad O–H stretching band near 3300 cm⁻¹ and characteristic Zn–Cl and C–O vibrations below 1000 cm⁻¹, confirming strong hydrogen‐bonding and polymer–ion interactions. In addition, the ^1^H NMR analysis (Figure 3b) further reveals chemical shift narrowing and redistribution in the 3.3–3.6 ppm region, indicating effective participation of PVA in modulating Zn^2^⁺ solvation through hydrogen bonding and steric confinement. Compared to DES, the polymer matrix in DEGE reduces excessive Zn^2^⁺–EG coordination and promotes a more uniform, de‐solvated ion environment, enabling enhanced structural stability and interfacial transport. These findings highlight DEGE as a robust, solid‐like electrolyte platform with improved ionic regulation and compatibility for neuromorphic and energy storage applications.

To further validate the electrochemical stability and controllability of DEGE, its phase transition process, water retention capability, and ion transport dynamics were systematically evaluated. The differential scanning calorimetry (DSC) analysis reveals that HGE exhibits a sharp exothermic peak at −39.78 °C with a high enthalpy release (ΔH = 90.45 J g^−1^), corresponding to water crystallization during cooling (Figure S13a, Supporting Information). In contrast, DEGE shows a weak endothermic transition at 58.77 °C (ΔH = −2.03 J g^−1^), likely associated with segmental chain relaxation or ionic dissociation. The absence of exothermic crystallization in DEGE confirms its superior thermal stability and freeze resistance, which is essential for reliable operation in variable‐temperature neuromorphic devices. As shown in Figure 3c, DEGE retains nearly 100% of its mass after 48 h under ambient conditions, significantly outperforming AE and PVA systems, which suffer from rapid evaporation or structural dehydration. This superior retention is consistent with the results of TG test in Figure S13b (Supporting Information), which can be attributed to the low‐volatility, highly hydrogen‐bonded network. As illustrated in Figure 3d, DEGE exhibits a high Zn^2^⁺ transference number of 0.87, indicating strong cation selectivity that favors directional ion migration, suppresses anion interference, and mitigates parasitic reactions during plating/stripping. Importantly, Figure S14 (Supporting Information) reveals a marked increase in the relaxation time constants and a reduction in total impedance based on distribution of relaxation time analysis. The enlarged time constants reflect slower, more regulated charge dynamics, which contribute to more stable conductance programming and operational uniformity in PSMs. To further analysis the conductance evolution trends, the conductance evolution trends of PSM using AE and DEGE were fitted by the double‐exponential decay model based on the Equation (2):^[^ ^25^, ^26^ ^]^

(2) $$G\left(t\right)=G_{1}\cdot \exp\left[-\left(\frac{t}{\tau_{1}}\right)\right]+G_{2}\cdot \exp\left[-\left(\frac{t}{\tau_{2}}\right)\right]+G_{0}$$

where *G*(*t*) represents the conductance at time *t*, *G*_1_ and *G*_2_ are the amplitudes corresponding to the fast and slow decay processes, τ_1_ and τ_2_ are the time constants that characterize the fast and slow relaxation processes, *G*_0_ is the baseline conductance value. This model captures the dual‐response behavior of the system, with representing the short‐term relaxation time and the long‐term recovery process. As shown in Figure 3e and Table S1 (Supporting Information), the conductance evolution behaviors during the plating (potentiation) process of Zn/AE/Cu and Zn/DEGE/Cu PSMs under different pulse voltages were investigated, with the data fitted using a double‐exponential decay model. Based on the fitting results, the Zn/AE/Cu PSM exhibits higher probability of conductance instability under pulse signal with a faster decay process (lower τ_1‐AE_ than τ_1‐DEGE_), whereas the Zn/DEGE/Cu PSM shows stronger stability during recovery, with a faster slow recovery process (lower τ_2‐DEGE_ than τ_2‐AE_). This phenomenon indicates that DEGE system is more suitable for applications requiring short‐term pulse conductance separability or long‐term conductance retention, particularly in low‐power tasks.

As shown in Figure 3f, the 3D surface profiles illustrate the morphological differences of Zn electrodeposition under AE (left) and DEGE (right) conditions. The Zn layer deposited in AE exhibits pronounced surface roughness with height variations exceeding 35 µm, along with severe dendritic protrusions, indicating significant localized nonuniformity and side reactions. In contrast, the Zn layer deposited with DEGE displays a much smoother surface with a maximum height fluctuation of only ≈18 µm, reflecting a more stable and uniform electrodeposition process. Figure S15 (Supporting Information) further corroborates this observation through SEM images. The Zn deposit from AE (Figure S15a, Supporting Information) shows a loosely stacked, porous lamellar structure, suggesting abundant defects and dendrite formation. In comparison, the Zn layer obtained in the DEGE electrolyte (Figure S15b, Supporting Information) exhibits a dense and fine‐grained morphology with good surface continuity, indicating that Zn^2^⁺ migration and reduction are more effectively regulated, facilitating the formation of a compact and uniform metal layer. Figure S16 (Supporting Information) provides further insight into the chemical composition of the plated Zn layer using DEGE. High‐resolution Zn 2p spectra (Figure S16b, Supporting Information) show that the dominant species is metallic Zn⁰, with only minor contributions from ZnO and Zn(OH)₂, confirming minimal surface oxidation. The quantified Zn^0^/Zn^2+^ ratio is as high as 3.78, indicating a highly stable metallic zinc layer with reduced oxidation. Moreover, the Cl 2p signal (Figure S16c, Supporting Information) is nearly undetectable, indicating a significant reduction or absence of Cl^−^ residues during plating, which can be attributed to the increased Zn^2^⁺ transference number and anhydrous properties in the DEGE, leading to a decrease in anion‐induced corrosion and suppression of Cl⁻ corrosion at the electrode surface. Collectively, these results highlight that the DEGE electrolyte significantly improves the smoothness, density, and chemical purity of Zn plating, effectively inhibiting dendrite growth and enhancing interfacial stability, thereby providing a robust material foundation for achieving high‐quality, low‐energy plating/stripping‐based memristive behavior.

**Figure 4:**
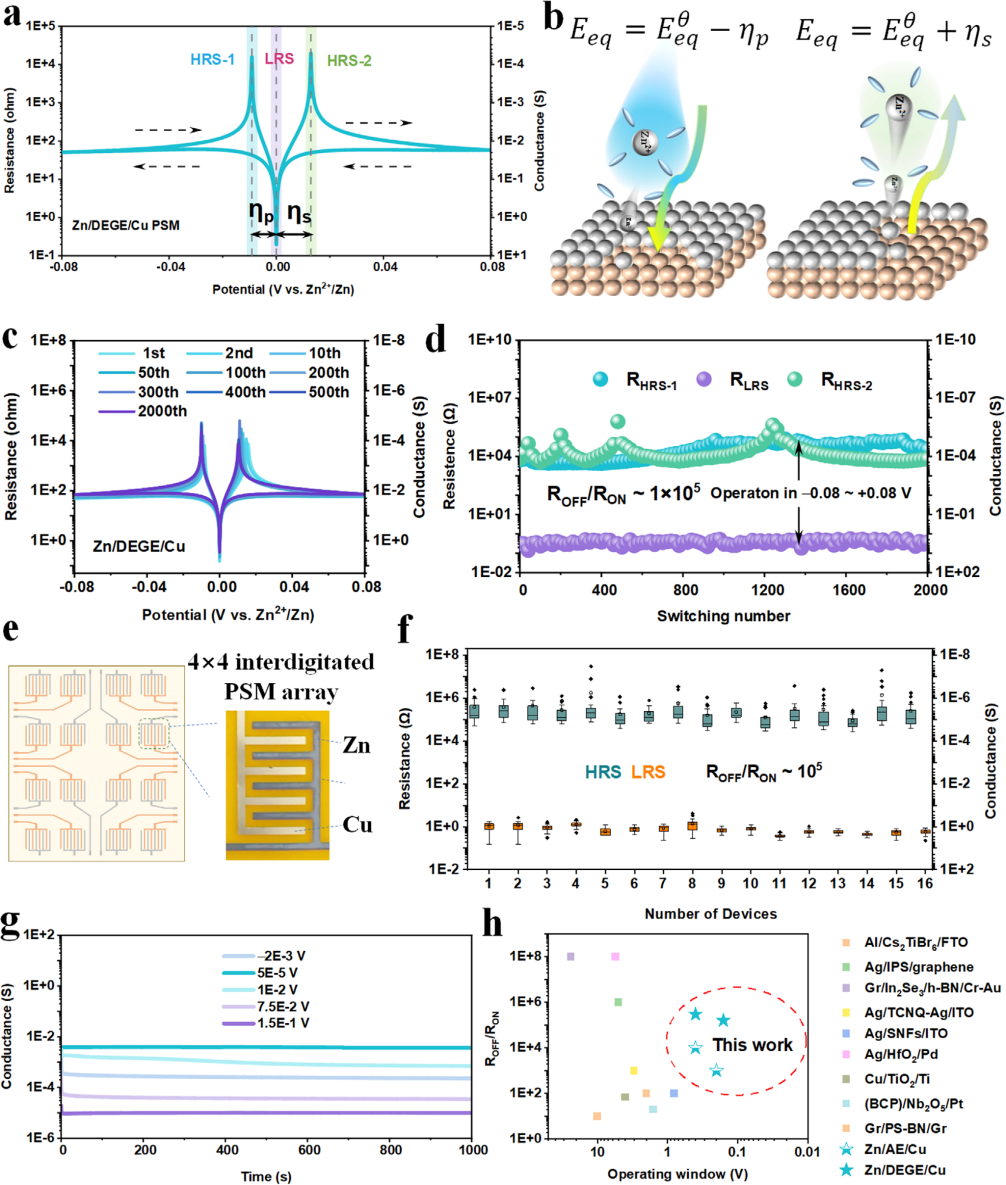
Reliability and biomimetic synaptic behavior of Zn/DEGE/Cu PSM. a) The Resistance/Conductance–Voltage curve of Zn/DGEG/Cu PSM. b) Schematic diagrams of deviation between ideal equilibrium potential $E_{eq}^{\theta }$ and experimental equilibrium potential *E_eq_* induced by polarization effect (η_*p*_ and η_*s*_) during plating/stripping. c) The Resistance/Conductance‐Voltage curves at different switching cycles. d) Endurance of LRS and HRS resistances with 2000 continuous DC switching cycles. e) The 4 × 4 interdigitated Zn/DEGE/Cu PSM array. f) Box plots of HRS and LRS distributions across the Zn/DEGE/Cu PSM array cells. g) Conductance retention under different applied voltages over 10^4^ s. h) The comparison of R_OFF_/R_ON_ and operating window between previous reports and this work.^[^ ^14^, ^27^, ^28^, ^29^, ^30^, ^31^, ^32^, ^33^, ^34^ ^]^

Owing to polarization‐induced deviations of the actual equilibrium potential *E_eq_* from $E_{eq}^{\theta }$ (*E_eq_* ≠ 0 *V* vs  · *Zn* ^2 +^  /*Zn*), a rare dual‐HRS behavior (HRS‐1 and HRS‐2) was observed during the negative and positive scans, respectively (**Figure** **4**a,b). Detailed analysis by integrating the plating/stripping thermodynamics with the electric double‐layer behavior at the DEGE/Cu interface was conducted to elaborate mechanism of the dual‐HRS phenomenon (Figures S17 and S18, Supporting Information). Specifically, the desolvation energy barriers and nucleation barriers contribute to a more negative actual *E_eq_* at V_HRS‐1_ than $E_{eq}^{\theta }$.^[^ ^35^ ^]^ Whereas the dissociation energy barrier for Zn‐Cu metal bonding and the energy barrier for Zn^2+^ to penetrate the electric double‐layer contribute to a more positive actual *E_eq_* at V_HRS‐2_ than $E_{eq}^{\theta }$ during stripping.^[^ ^36^, ^37^, ^38^ ^]^ As shown in Figure 4c,d, both the switching thresholds and non‐volatile states remain highly stable even after 2000 cycles, underscoring the excellent reversibility and durability of the DEGE system. In addition, dual‐HRS behavior is symmetrically located ≈−10.0 mV and +11.1 mV, respectively. This also leads to the LRS appearing at ≈0 V vs Zn^2+^/Zn (*V_LRS_* = 15.3 µV) of Zn/DEGE/Cu PSM, highlighting the self‐activated nature of the conductive transition with negligible energy input (Figure S19, Supporting Information). Furthermore, even after one week of ambient storage, the Zn/DEGE/Cu PSM maintains a stable resistance window over 2000 switching cycles (Figure S20, Supporting Information).

A flexible 4×4 Zn/DEGE/Cu PSM array was fabricated via electrochemical plating onto an interdigitated electrode layout, as shown in Figure 4e. The device array exhibits a highly uniform structural configuration, with well‐defined electrode spacing and encapsulated DEGE electrolyte coverage ensuring mechanical integrity. Figure 4f and Figure S21 (Supporting Information) present the statistical distribution of the HRS and LRS across all 16 devices. Consistent switching ratio and switching voltage are achieved with minimal device‐to‐device variation, indicating excellent switching uniformity and reproducibility. These results validate the scalability and integration potential of the Zn/DEGE/Cu PSM system for large‐area neuromorphic hardware platforms. To further explore the dynamic plasticity of the Zn/DEGE/Cu PSM device, spike‐rate dependent plasticity (SRDP) behaviors under varying pulse parameters were evaluated. As shown in Figure S22a (Supporting Information), an increase in pulse duration *t*_1_​ leads to a progressive enhancement in conductance change ΔG, suggesting stronger synaptic potentiation with longer electrochemical stimulation time. This reflects a rate‐strengthened learning process, where prolonged ion migration and plating under extended pulses enhance the device's conductance response. In contrast, Figure S22b (Supporting Information) reveals that as the pulse interval *t*_2_ increases, ΔG gradually decays, indicating a loss of temporal summation and a tendency toward synaptic depression. These behaviors mimic biological forgetting and fatigue, where sparse or delayed inputs fail to maintain elevated synaptic weights. The LTP/LTD behaviors tuned by voltage pulses were also observed based on Zn/DEGE/Cu PSM as shown in Figure S23 (Supporting Information).Together, these results demonstrate that the Zn/DEGE/Cu PSM supports both short‐term potentiation and depression modulated by spike rate and timing, establishing a solid foundation for hardware‐based temporal learning.

The long‐term stability and ultra‐low power performance of the Zn/DEGE/Cu PSM device were further validated through retention and benchmarking analysis. As shown in Figure 4g, under various applied biases ranging from −2 to 150 mV, the device maintains remarkably stable conductance levels over 1000 s, with negligible drift across multiple conductance states. This demonstrates the excellent non‐volatility and temporal retention of the Zn/DEGE/Cu PSM, even under sub‐threshold bias conditions—ideal for standby neuromorphic operations. Figure 4h presents a benchmarking plot comparing the *R_OFF_*/*R_ON_* ratio and operating window of the Zn/DEGE/Cu device against other reported memristive systems. In summary, the proposed PSM shows strong potential to compete with state‐of‐the‐art oxide, organic, and electrochemical memristive technologies with a millivolt‐level operating voltage, a high switching ratio (>10⁵), and suitability for energy‐efficient and short/long‐term neuromorphic operations (Table S2, Supporting Information).

**Figure 5:**
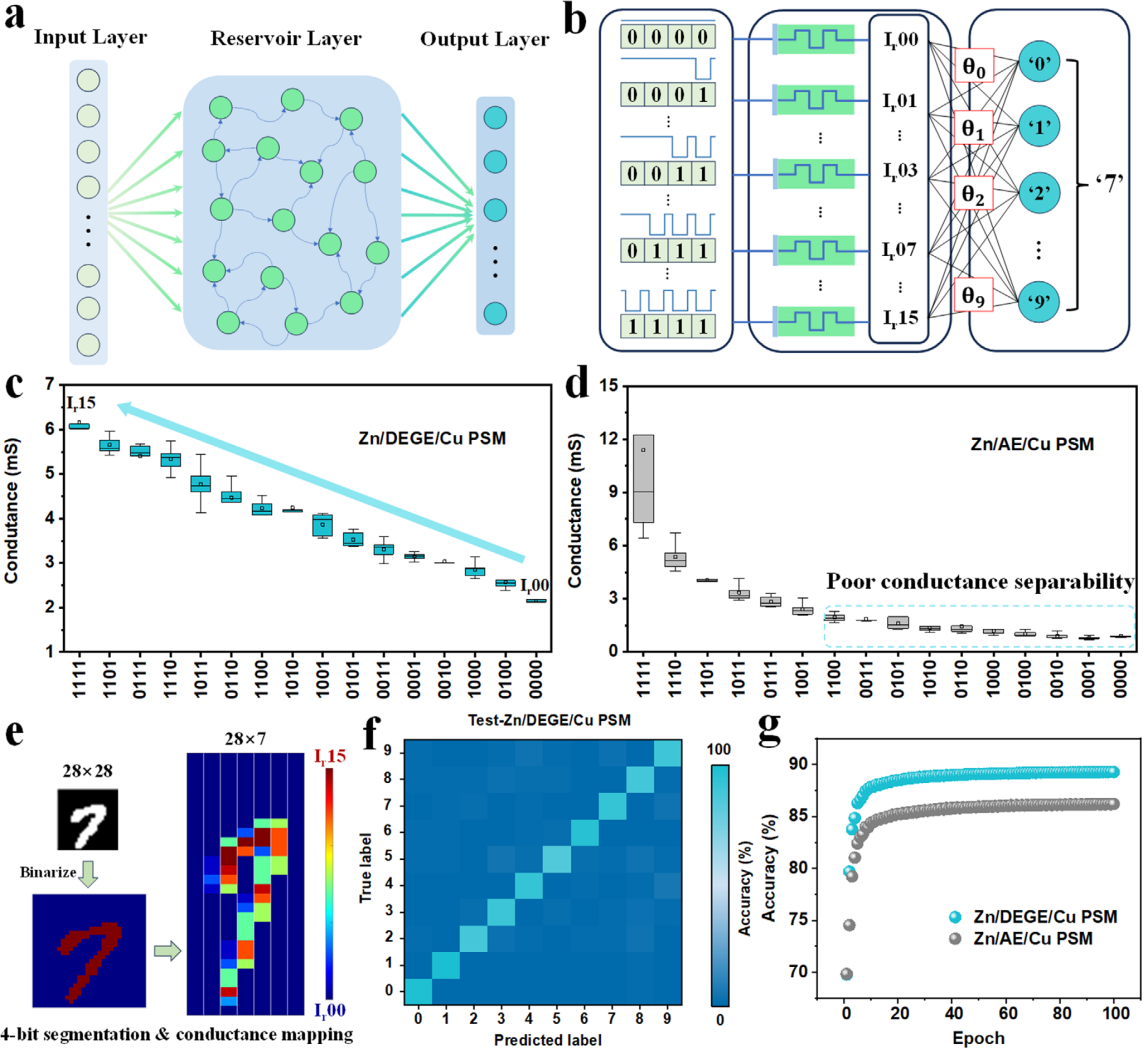
Reservoir computing based on PSM. a) Reservoir computing architecture. b) Implementation of handwritten character recognition task (Modified National Institute of Standards and Technology database, MNIST) based on 4‐bit pulse coding. Readout conductance after 4‐bit encoded pulses of c) Zn/DEGE/Cu PSM array, and d) Zn/AE/Cu PSM array. e) Schematic diagram of binarization and conductance mapping process of handwritten image. f) Confusion matrix of test set for reservoir computing encoded by Zn/DEGE/Cu PSM array (10 000 handwritten digits). g) Recognition accuracy of reservoir computing encoded by Zn/DEGE/Cu PSM array and Zn/AE/Cu PSM array.

To exploit the intrinsic temporal dynamics and analog programmability of electrochemical memristors, a reservoir computing (RC) framework was established using Zn/Cu PSM.^[^ ^39^, ^40^, ^41^ ^]^ As illustrated in **Figure** **5**a, the RC system comprises three components: an input layer that encodes external stimuli into voltage pulse streams, a non‐linear reservoir layer emulated by the interconnected PSM network, and an output layer responsible for classification. A 4‐bit temporal pulse coding scheme was applied (Figure 5b; Figure S24, Supporting Information), transforming pixel‐level grayscale data from MNIST images into sequential electrical stimuli. Each 4‐bit pulse combination modulates the conductance of individual PSM devices, enabling analog encoding of local spatial features from the input images. Importantly, the Zn/DEGE/Cu PSM array demonstrates a quasi‐linear and well‐separated 16‐level conductance distribution (Figure 5c), in stark contrast to the Zn/AE/Cu PSM using conventional aqueous electrolyte, which suffers from poor conductance discriminability and high variability (Figure 5d).

This enhanced conductance precision significantly improves the digit recognition performance of the RC system. As depicted in Figure 5e, the 28 × 28 MNIST images were first binarized and segmented into 4‐bit encoded 28 × 7 pulse sequences for direct mapping to the PSM conductance domain. Impressively, the Zn/DEGE/Cu PSM exhibits faster loss convergence and achieves a lower steady‐state error during training (Figure S25, Supporting Information). In contrast, the Zn/AE/Cu PSM suffers from poor conductance separability, leading to cumulative errors in the readout layer (Figure S26, Supporting Information). The quasi‐linear conductance response enabled by the DEGE electrolyte ensures more stable learning dynamics and improved generalization performance. The resulting confusion matrix (Figure 5f) demonstrates a highly diagonalized response with lower, confirming accurate classification across all digit classes using the Zn/DEGE/Cu‐based RC platform. The comparative training curves in Figure 5g further underscore the benefit of superior conductance separability: the Zn/DEGE/Cu PSM system reaches 89.3% recognition accuracy within 100 epochs, outperforming the Zn/AE/Cu system. These results validate the advantage of the DEGE‐assisted Zn/Cu electrochemical interface in achieving stable, multi‐level analog modulation, thereby enabling reliable information processing in neuromorphic hardware.

## 3 Conclusion

A Zn/DEGE/Cu PSM is developed, exhibiting stable, low‐voltage, and bio‐inspired conductance switching behavior. Unlike conventional devices that rely on conductive filaments or oxygen vacancies, the switching process is governed exclusively by the reversible migration and redox reactions of Zn^2^⁺, enabling greater material tunability and synaptic emulation fidelity. The introduction of DEGE provides a mechanically flexible and chemically robust ionic medium that suppresses dendrite formation and parasitic reactions while promoting uniform Zn plating. The device supports switching with a low‐resistance state centered at 15.3 µV and displays symmetric dual high‐resistance states at −10.0 mV and +11.1 mV, regulated by actual equilibrium potential, with excellent endurance maintained over 2000 cycles. Key neuromorphic functionalities, including SRDP and LTP/LTD behavior modulated by pulse width, interval, and repetition, are reliably reproduced. Notably, a performance comparison reveals a favorable balance between ultra‐low operating voltage (< 0.1 V) and high resistance contrast (*R_OFF_*/*R_ON_* ≈10⁵). When implemented in a reservoir computing architecture for temporal pattern recognition, the Zn/DEGE/Cu PSM achieves rapid training convergence and high accuracy (89.3%) based on the stable multi‐level conductance encoding, revealing PSM as an important direction for scalable, energy‐efficient neuromorphic computing.

## Conflict of Interest

The authors declare no conflict of interest.

## Supporting information



Supporting Information

## Data Availability

The data that support the findings of this study are available from the corresponding author upon reasonable request.
