# Supplementary material for: Ultra‐Low Operating Voltage Memristors Based on Plating/Stripping Reactions
Source: Adv Sci (Weinh). 2025 Jul 21;12(39):e10370. doi: 10.1002/advs.202510370 (PMC12533213; doi:10.1002/advs.202510370)
Supplement: Supplementary file 1 — Supporting Information [file ADVS-12-e10370-s001.pdf]

ADVANCED SCIENCE Open Access

Supporting Information

for *Adv. Sci.*, DOI 10.1002/advs.202510370

Ultra-Low Operating Voltage Memristors Based on Plating/Stripping Reactions

Lingbo Yao, Zhurui Wang, Yanyu Sun, Xiaowei Chi and Yu Liu**

Supporting Information

Ultra-low Operating Voltage Memristors based on Plating/Stripping Reactions

Lingbo Yao[1,2], Zhurui Wang[1,2], Yanyu Sun[1,2], Xiaowei Chi[1*], Yu Liu[1*]

[1]Shanghai Institute of Ceramics, Chinese Academy of Sciences

Shanghai 200050, China

[2]University of Chinese Academy of Sciences

Beijing 100049, China

*Email: xwchi@mail.sic.ac.cn; yuliu@mail.sic.ac.cn

EXPERIMENTAL SECTION

Fabrication of Zn-, Cu-, and Na-based PSM

The Zn, Cu, and Ti metal foils were first mechanically polished using fine-grade sandpaper to remove surface oxides and impurities. The polished electrodes were then briefly immersed in diluted acid, rinsed thoroughly with deionized water, ultrasonicated in anhydrous ethanol, vacuum-dried, and subsequently cut into circular discs for use as active electrodes. Commercial sodium metal electrodes were used without further treatment. As a proof of concept, Zn, Cu, and Na were individually employed as the electrochemically active plating/stripping electrodes. Glass fiber separators were used in combination with three types of electrolytes: 3 M $ZnSO_4$ aqueous solution (AE) or DEGE for Zn-based systems, saturated $CuSO_4$ aqueous solution for Cu-based systems, and 1 M $NaClO_4$ in propylene carbonate for Na-based systems. Cu, Ti, and Cu foils were used as the respective conductive substrate electrodes. The full device stack was assembled in a sandwich-type configuration and sealed with acrylic resin after electrical lead-out from the Ti current collector.

Preparation of DES, DEGE, and HGE

To prepare the DEGE, anhydrous $ZnCl_2$ and ethylene glycol (EG) were mixed at a molar ratio of 1:4 and stirred magnetically at 80 °C for 12 h to form a homogeneous deep eutectic solvent (DES). Polyvinyl alcohol (PVA, 1799, 10 wt%) was then dissolved into the DES under magnetic stirring at 90 °C until the mixture became completely transparent. The resulting solution was cast into a polytetrafluoroethylene mold and subjected to freeze-induced crosslinking at –20 °C for 1 h to obtain DEGE. For the hydrogel electrolyte (HGE), an aqueous PVA hydrogel (10 wt%) was prepared via three freeze-thaw cycles and subsequently immersed in 3 M $ZnCl_2$ aqueous solution for 5 min to complete ion incorporation.

Fabrication of Zn/DEGE/Cu PSM arrays.

For the fabrication of Zn/DEGE/Cu PSM arrays, a flexible polyimide substrate was used. Interdigitated copper electrode patterns were defined on the substrate via hot-pressing, and etching processes. Prior to metal deposition, the DEGE precursor solution was drop-cast onto the patterned electrodes and subjected to freeze-induced

crosslinking at –20 °C for 1 h to form the gel electrolyte in situ. Zinc foil was then used as the counter electrode, and galvanostatic electrodeposition was performed for 1 h. After removing the zinc foil, integrated Zn/DEGE/Cu PSM arrays were obtained.

Materials Characterization

The thermal properties of hydrogels were characterized by a thermogravimetric analyzer (TGA, NETZSC, STA 409 PC) with a heating rate of 5 °C min^{-1} in the N_2 atmosphere. Characteristic functional groups and bond structures were analyzed by the attenuated total reflection Fourier transform infrared spectroscopy (ATR-FTIR, Thermo Scientific Nicolet iS5) in the range of 4000 to 400 cm^{-1} with the resolution of 4 cm^{-1}. The ^{1}H NMR spectra were characterized using the heavy water as a deuterated reagent (Bruker Avance NEO 400MHz). Morphology and roughness of the Cu electrodes were analyzed by optical profiler (Mahr LD130). Scanning electron microscopy (SEM, JEOL, JSM-6510) was employed to observe the morphology of the Cu electrodes. The electronic structure and corresponding composition were characterized by X-ray photoelectron spectroscopy (XPS, Thermo Scientific K-Alpha).

Electrical Measurements

Electrochemical impedance spectroscopy (EIS), and cyclic voltammetry (CV), Chronoamperometry (CA) were performed on the electrochemical workstation (Autolab instrument PGSTAT302N). Galvanostatic charge/discharge (GCD) cycling tests were carried out on the LAND CT2100A. The CV tests were performed with a scan rate of 2-100 mV s^{-1}. The Zn transference number ($t_{Zn^{2+}}$) test was tested based on CA test. At a polarization voltage, the $t_{Zn^{2+}}$ was calculated based on the Equation (1):

$$t_{Zn^{2+}} = \frac{I_S(\Delta V - I_0 R_0)}{I_0(\Delta V - I_S R_S)} \qquad (1)$$

where I_0 and I_s are the initial and steady-state current, ΔV is the applied constant potential, and R_0 and R_s are the initial and steady-state interface resistance, respectively. For the EIS tests, the AC signal ranging from 0.01 Hz to 1000 kHz was used for all the EIS tests. The DRT analysis was performed by using DRT Tools[1]. The differential capacitance curve was calculated from the Equation (2):

$$C = -(\omega Z_{im})^{-1} \qquad (2)$$

where C is the differential capacitance and ω is the angular frequency, Z_{im} is the imaginary part of the impedance, and 1000 Hz was selected as the specific frequency.

Reservoir computing

A reservoir computing model based on a memristor array and 4-bit pulse signals was constructed for MNIST handwritten digit recognition. The original 28×28 grayscale images were binarized using a fixed threshold and segmented row-wise into 196 groups of 4-bit pulse sequences. Each 4-bit pattern was mapped to one of 16 experimentally measured conductance levels of the memristor device, forming a 28×7 readout current matrix that was then flattened into a one-dimensional feature vector. All features from the training (60,000 samples) and testing (10,000 samples) sets were standardized before being fed into a one-vs-all logistic regression classifier. The classifier was trained using the fmincg function over 100 iterations, during which the weight vectors for all 10-digit classes were updated and the loss values recorded at each epoch. The model's classification performance and temporal encoding stability were evaluated through confusion matrices and accuracy metrics on both training and testing sets.

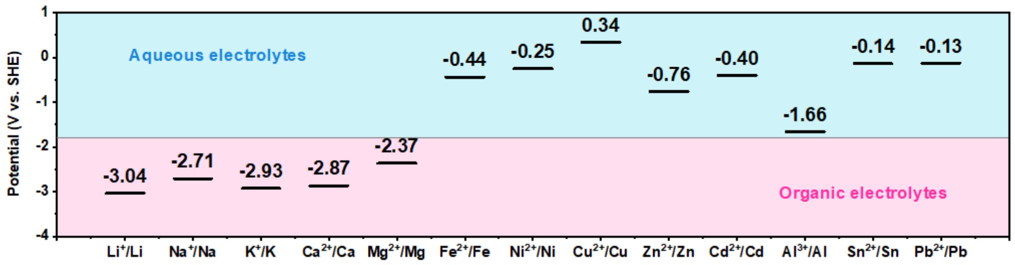

Figure S1. Standard electrode potentials of common metal electrodes in plating/stripping reactions.

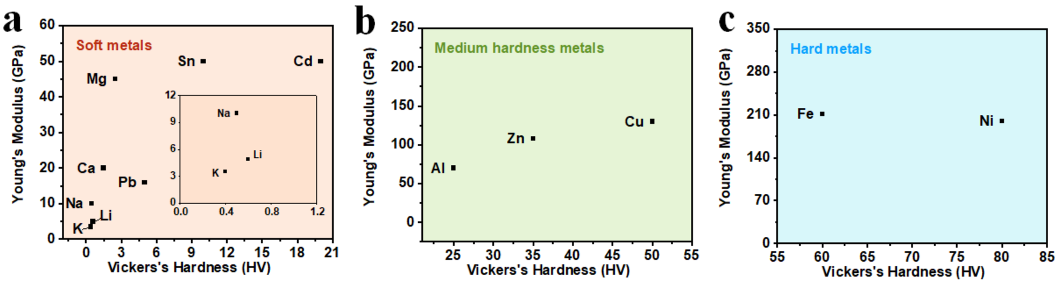

Figure S2. Comparison of Young's modulus and Vickers's hardness of common metal electrodes based on the classification of (a) Soft metals, (b) Medium hardness metals, and (c) Hard metals.

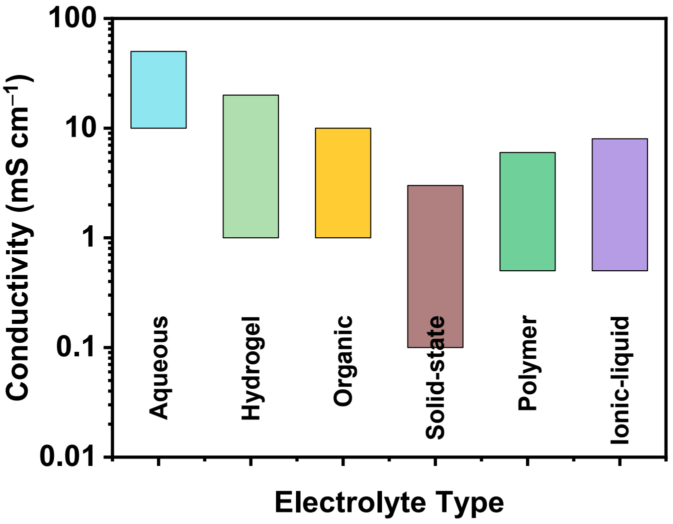

Figure S3. Ionic conductivity ranges of common types of electrolytes.

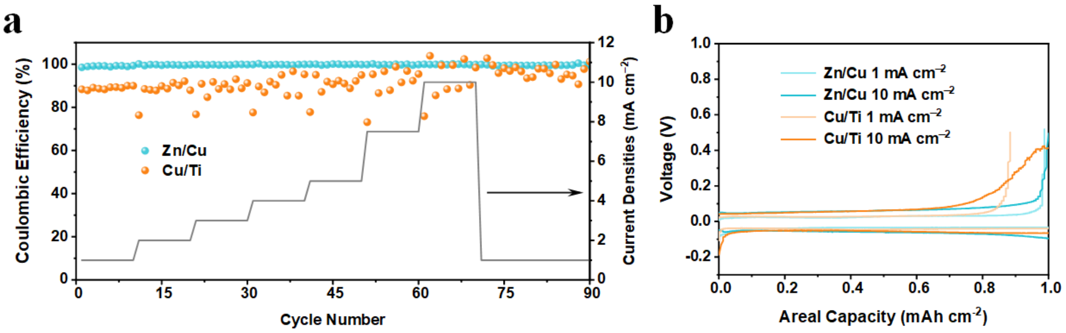

Figure S4. (a) Comparison of Coulombic efficiency of Zn/Cu and Cu/Ti PSMs at different current densities. (b) Voltage-Areal capacity curves of Zn /Cu and Cu /Ti PSMs at current densities of 1 and 10 mA cm^{-2}.

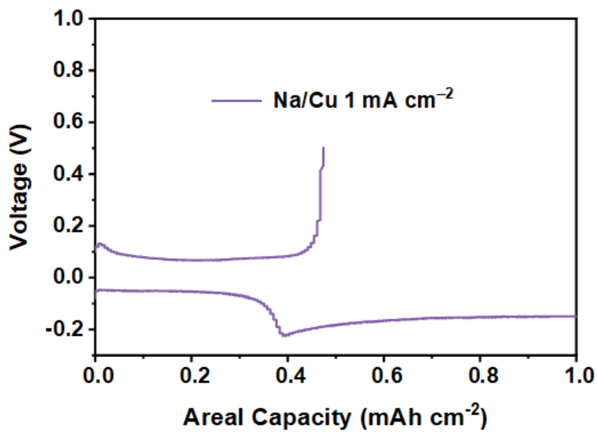

Figure S5. Voltage-Areal capacity curves of Na/Cu PSM at current densities of 1 mA cm^{-2}.

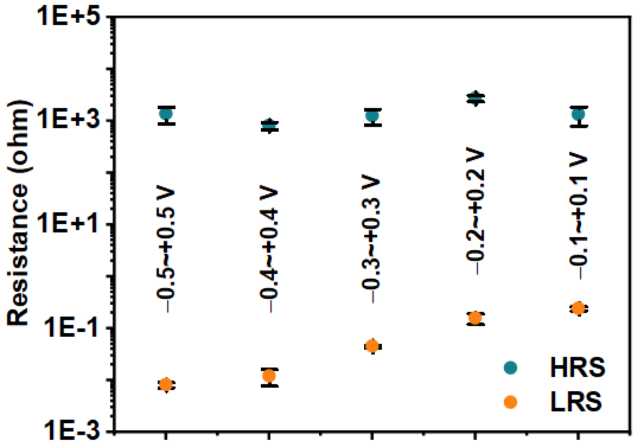

Figure S6. R_{OFF}/R_{ON} of Zn/AE/Cu PSM under different operating windows.

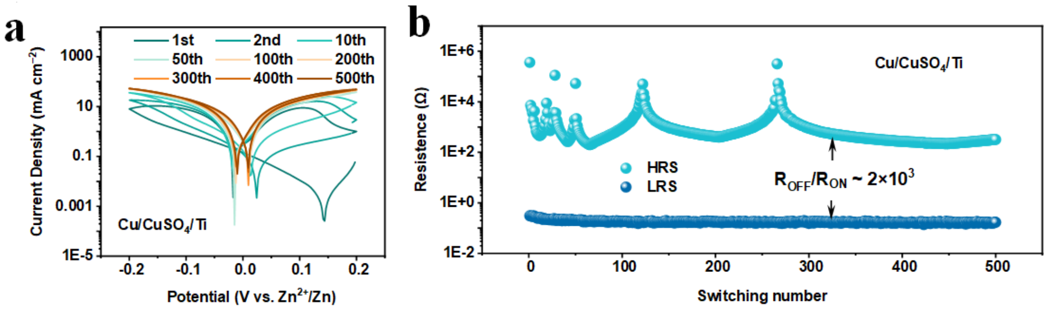

Figure S7. (a) The switching of the Cu/Ti PSM. (b) Endurance of LRS and HRS resistances with 500 continuous DC switching cycles.

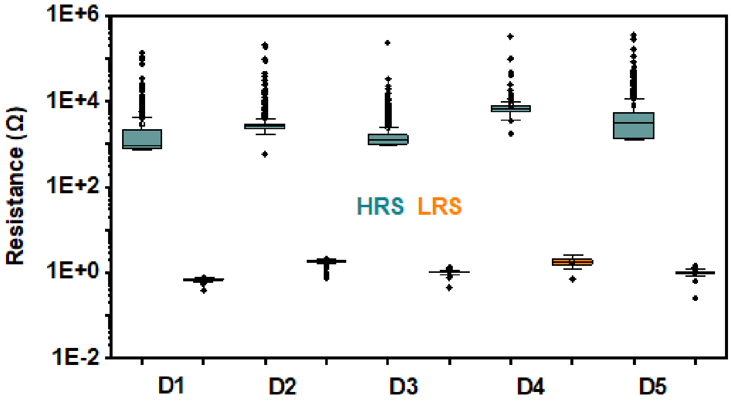

Figure S8. Box plots depicting the distribution of HRS and LRS across the Zn/AE/Cu PSM devices.

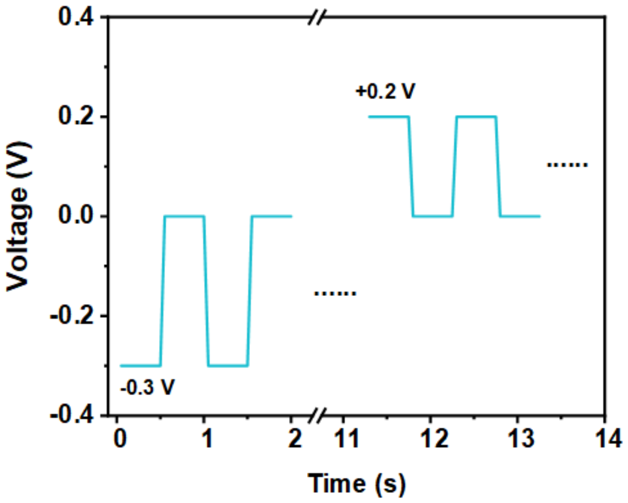

Figure S9. The waveform of the applied pulsed voltage for LTP/LTD test of Zn/AE/Cu PSM.

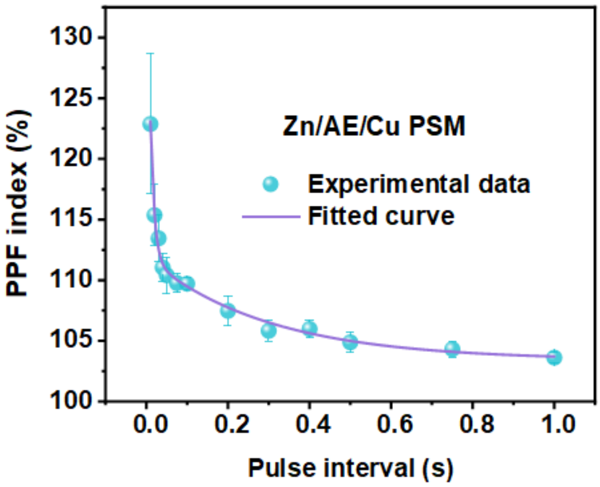

Figure S10. The PPF (defined as $(A_2\text{-}A_1)/A_1 \times 100\%$) index versus pulse interval by applying a paired pulse.

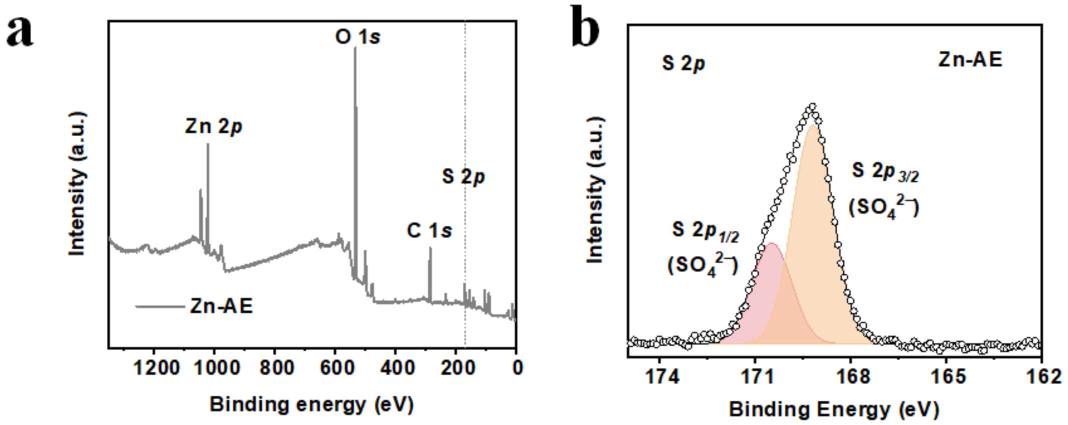

Figure S11. The (a) XPS survey and (b) S 2*p* of substrate electrode with Zn plating layer using AE.

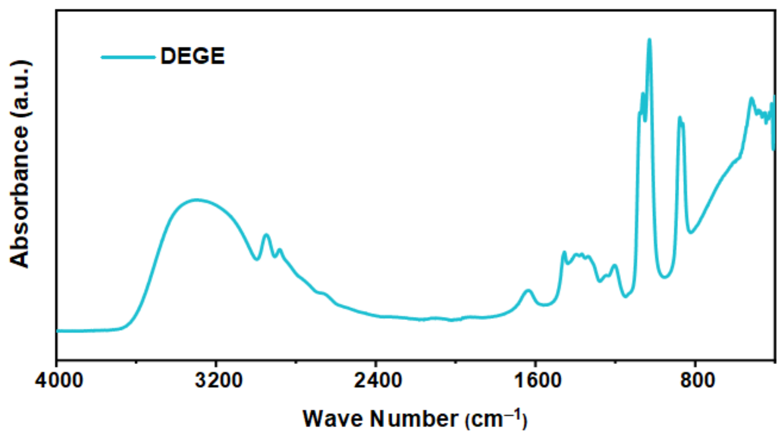

Figure S12. The ATR-FT-IR spectra of DEGE.

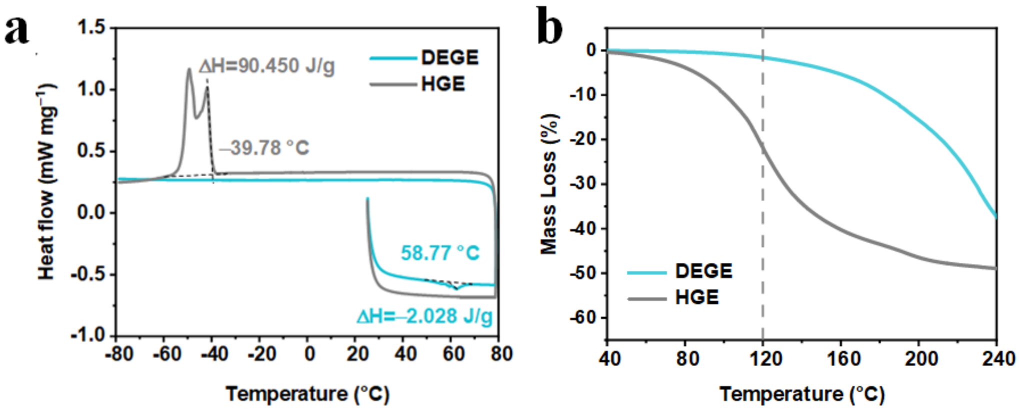

Figure S13. Thermal analysis of DEGE and HGE. (a) DSC tests. (b) TG tests.

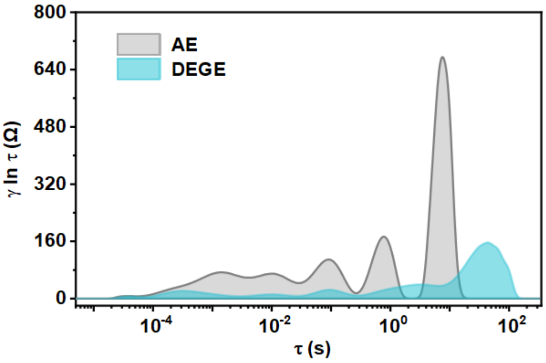

Figure S14. The DRT spectra of different interface using AE and DEGE.

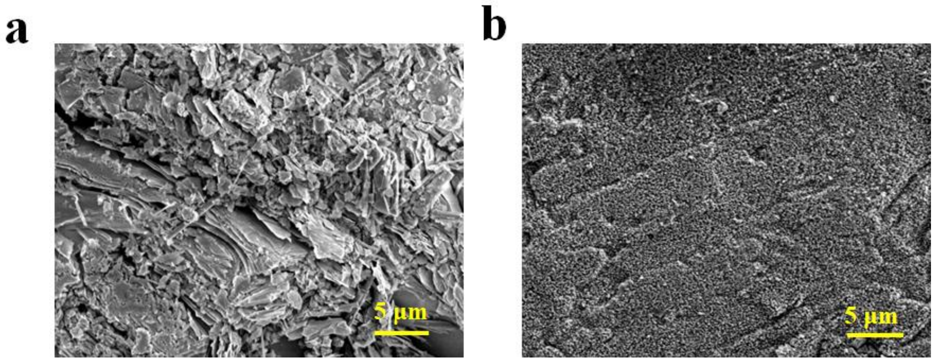

Figure S15. SEM images of substrate electrode with Zn plating layer using (a) AE, and (b) DEGE.

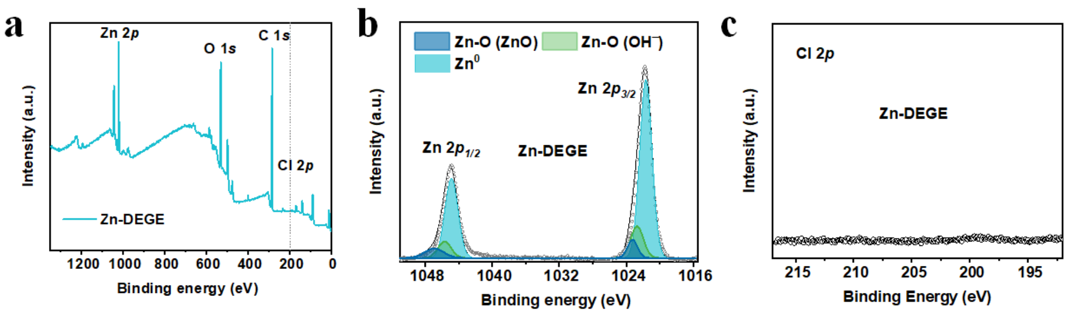

Figure S16. The (a) XPS survey, (b) Zn 2*p*, and (c) Cl 2*p* spectra of substrate electrode with Zn plating layer using DEGE.

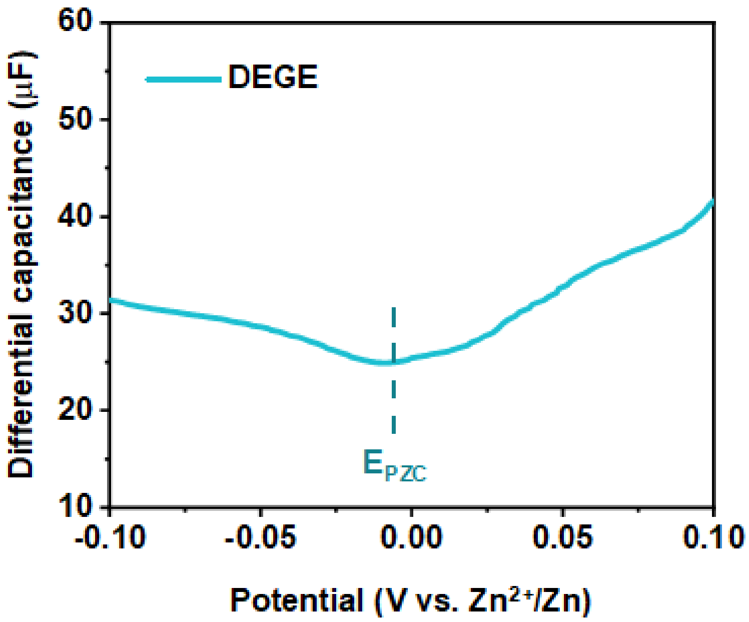

Figure S17. Differential capacitance–potential curves of the Zn/DEGE/Cu PSM, showing asymmetric charge accumulation behavior near 0 V (vs. Zn^{2+}/Zn).

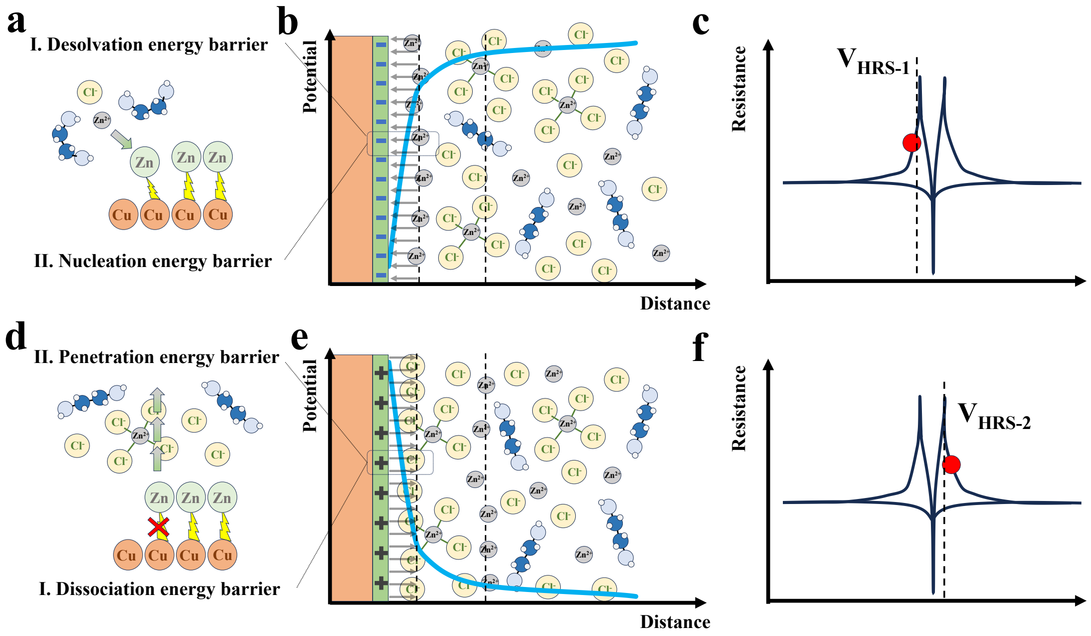

Figure S18. The illustration diagram of (a) main energy barrier during plating reaction and corresponding (b) electric double-layer of DEGE/Cu interface at potentials slightly more negative than V_{HRS-1} (c). The illustration diagram of (d) main energy barrier during stripping reaction and corresponding (e) electric double-layer of DEGE/Cu interface at potentials slightly more positive than V_{HRS-2} (f).

As elaborated by the differential capacitance curves in Figure S17 and the mechanistic diagram in Figure S18, further analysis was conducted by integrating the reaction mechanism with the electric double-layer behavior at the DEGE/Cu interface.

Specifically, during the plating process, the reduction of Zn^{2+} to Zn^0 requires overcoming both desolvation energy barriers and nucleation barriers, resulting in a more negative actual equilibrium electrode potential at V_{HRS-1}[2]. Conversely, during the stripping process, the high lattice affinity between the Zn^0 and Cu substrate electrode leads to alloying tendencies[3,4]. This necessitates additional dissociation energy to break the Zn-Cu metallic bonds. Furthermore, the differential capacitance curve exhibits distinctly asymmetric potential-dependent evolutionary behavior. The potential range above the point of zero charge (PZC) indicates significant specific adsorption of anionic species forming a compact thin double layer, which may create steric hindrance effects that impede Zn^{2+} diffusion[5]. As a result, the strong dissociation energy barrier

of Zn-Cu metallic bonds coupled with the subsequent penetration energy barrier for Zn^{2+} crossing the anion adsorption layer causes the actual equilibrium electrode potential during stripping (V_{HRS-2}) to become more positive. In summary, the emergence of dual high-resistance states can be attributed to the combined influence of double-layer behavior and reaction thermodynamics.

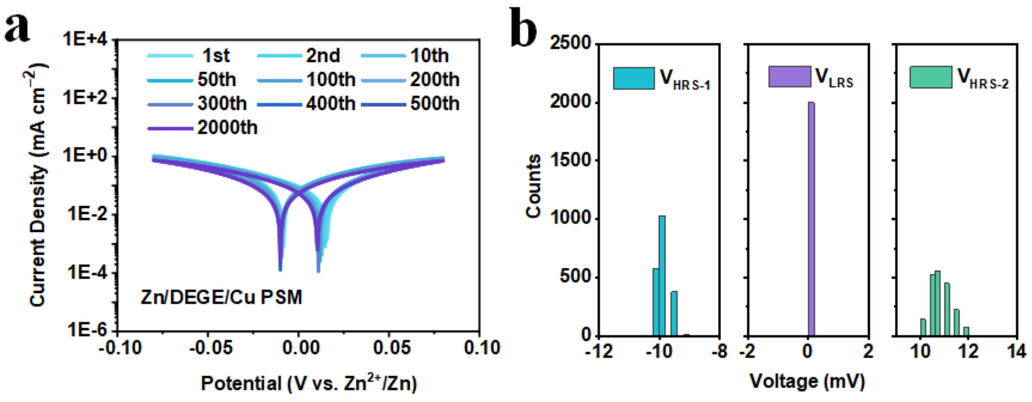

Figure S19. Stability and interfacial electric double-layer behavior of Zn/DEGE/Cu PSM. (a) The I-V curves of Zn/DGEG/Cu PSM at different switching cycles. (b) Histogram statistics of the voltages corresponding to HRS and LRS of Zn/DEGE/Cu PSM.

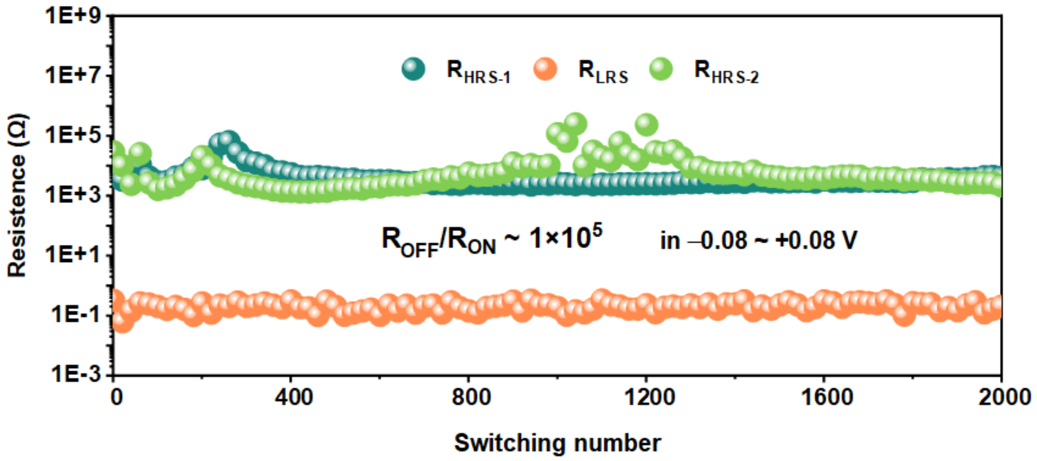

Figure S20. Endurance of LRS and HRS resistances with 500 continuous DC switching cycles using Zn/DEGE/Cu PSM after standing still for one week.

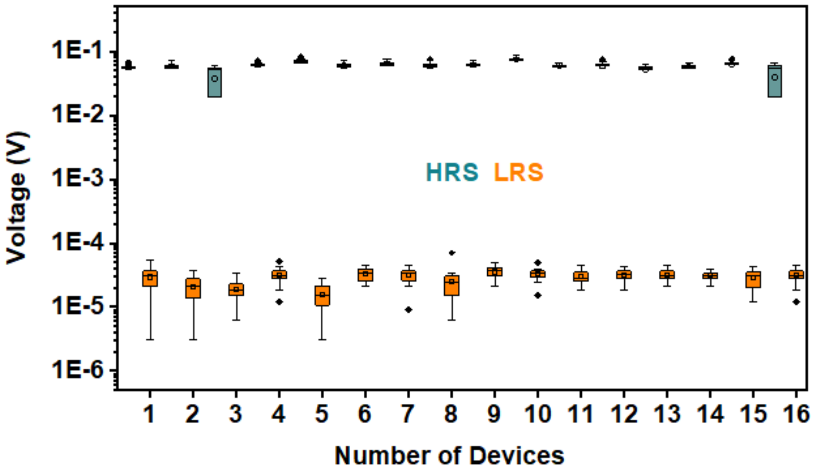

Figure S21. Box plots of voltage of HRS and LRS distributions across the 16 Zn/DEGE/Cu PSM array cells.

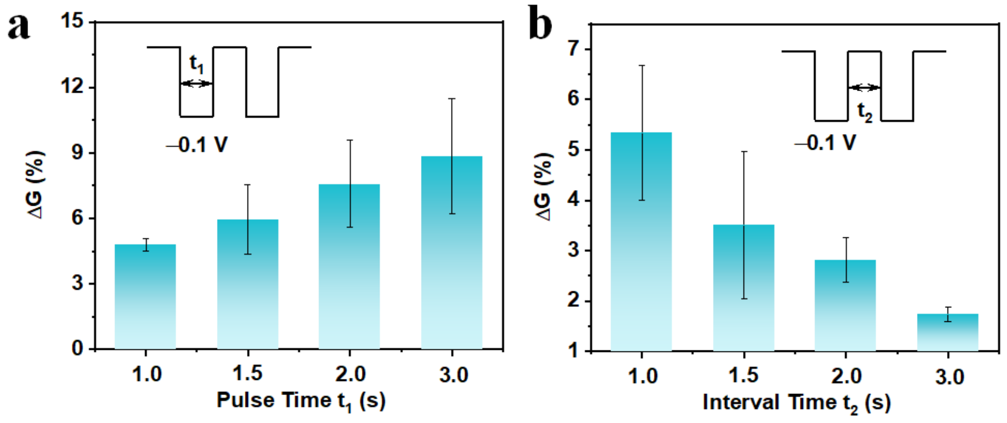

Figure S22. SRDP behaviors of Zn/DEGE/Cu PSM with different (a) pulse time, and (b) interval time.

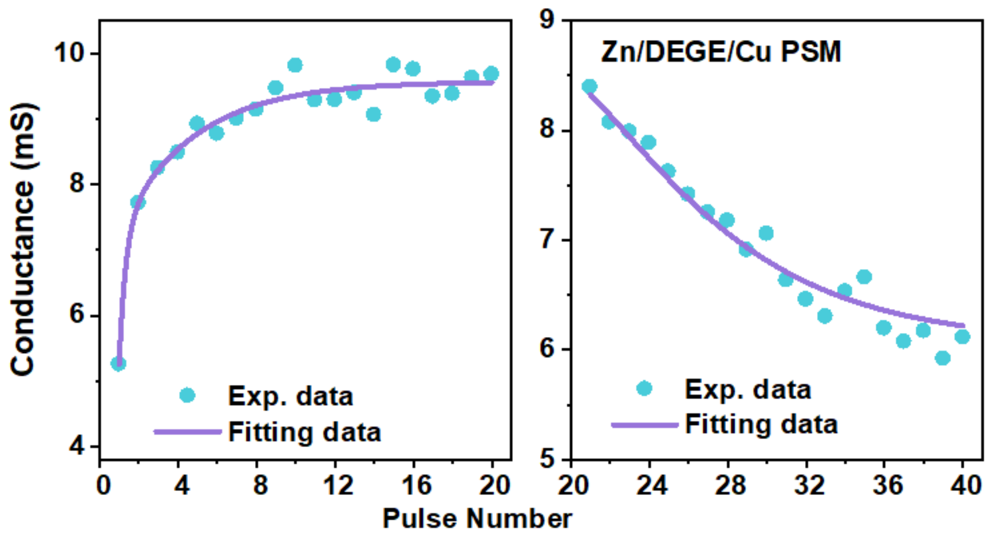

Figure S23. LTP/LTD behavior of Zn/DEGE/Cu PSM.

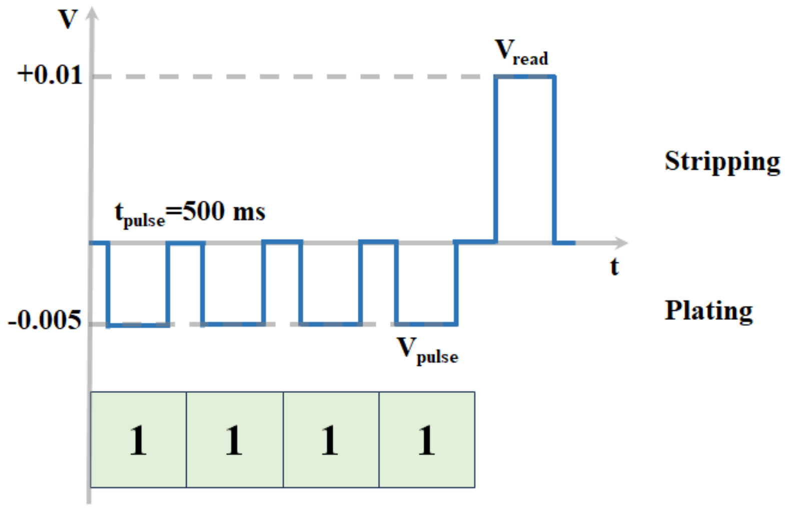

Figure S24. The waveform of the applied pulsed voltage for 4-bit pulse encoding ‘1111’ of Zn/AE/Cu PSM.

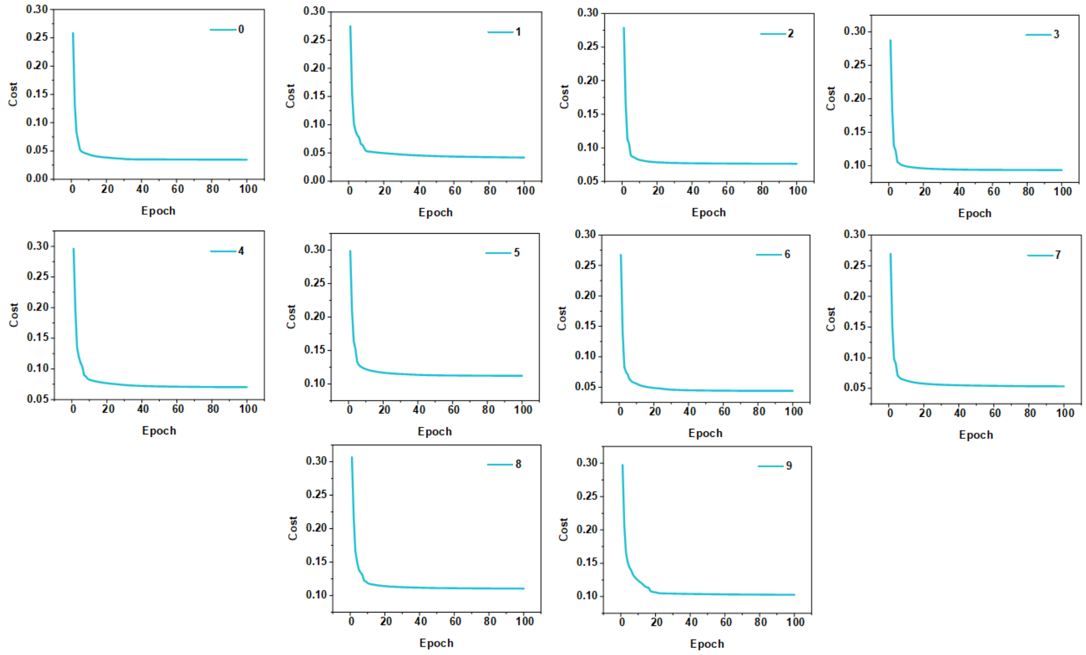

Figure S25. Loss function of processing ten handwritten digits using Zn/DEGE/Cu PSM-encoded pulse inputs.

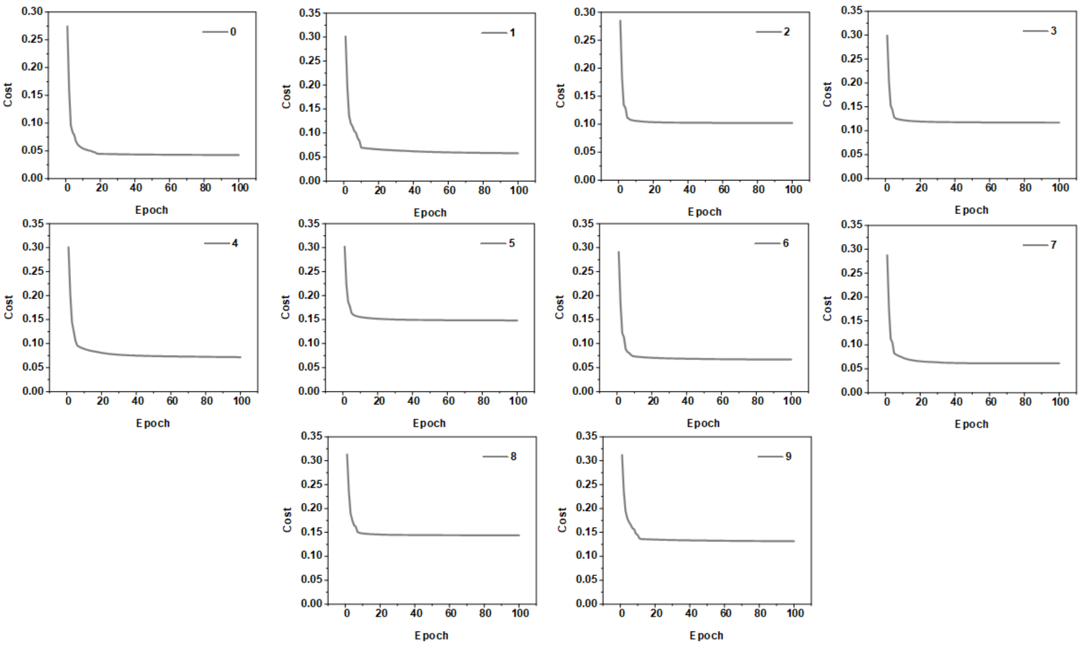

Figure S26. Loss function of processing ten handwritten digits using Zn/AE/Cu PSM-encoded pulse inputs.

Table S1. The time constants fitted using double exponential model during the plating process for various pulse voltages used.

**Pulse voltage (V)**	$\tau_{1\text{-AE}}$ **(s)**	$\tau_{2\text{-AE}}$ **(s)**	$\tau_{1\text{-DEGE}}$ **(s)**	$\tau_{2\text{-DEGE}}$ **(s)**
-0.005 V	0.0253	1.5094	0.0555	1.0518
-0.05 V	0.0068	0.5310	0.0575	0.4009
-0.1 V	0.0633	0.2637	0.0778	0.2340
-0.2 V	0.0450	0.2572	0.0509	0.1450

Table S2. Comparison of Zn/DEGE/Cu PSM against state-of-the-art oxide, organic, and electrochemical memristors in terms of operating voltage, R_{OFF}/R_{ON}, retention time, endurance cycles, and synaptic behavior.

Device structure	Operating voltage	Endurance cycles	R_{on}/R_{off}	Retention time	Synaptic behavior
**Zn/DEGE/Cu**	**0.16 V**	**2000**	**10^5**	**>10^3 s**	**LTP/LTD, SRDP, PPF**
Cu/h-BN/Au[6]	1.40 V	-	10^2	10^4 s	LTP/LTD
Ag/h-BN/Au[7]	0.27 V	300	10^4	10^6 s	-
Ta-TaS_xO_y-TaS_2[8]	7 V	-	10^5	-	STP/LTP, SRDP, PPF
Ag/Al_2O_3/Ta_2O_5/Pt[9]	4 V	10000	10^2	10^5 s	-
Pt/Al_2O_3/TiO_{2-x}/Ti[10]	2 V	5000	10^4	5×10^4 s	-
Ag/IPS/graphene[11]	4.5 V	1000	10^8	10^4 s	LTP/LTD
ITO/TCNQ-Ag/Ag[12]	3 V	80	10^3	10^4 s	LTP/LTD
Al/Ta-Cu_3COF/ITO[13]	2 V	40	-	2×10^3 s	PPF/PPD, STDP
Cu/PEDOT:PSS-PP:PVDF/Ti[14]	2 V	100	7	-	-
Au/ PBDTT-BQTPA/ITO[15]	1.7 V	10^8	10	10^4 s	-
ZIF-7/1 M KOH/YP-50F[16]	2.5 V	100	10	2.5×10^4 s	LTP/LTD
PC Å-scale channels[17]	2 V	-	2	-	LTP/LTD, PPF/PPD, STDP
ICP nanofluidic memristors[18]	30 V	15	5	-	-

Reference

[1] T. H. Wan, M. Saccoccio, C. Chen, F. Ciucci, *Electrochim. Acta* **2015**, *184*, 483.

[2] X. Yu, M. Chen, Z. Li, X. Tan, H. Zhang, J. Wang, Y. Tang, J. Xu, W. Yin, Y. Yang, D. Chao, F. Wang, Y. Zou, G. Feng, Y. Qiao, H. Zhou, S.-G. Sun, *J. Am. Chem. Soc.* **2024**, *146*, 17103.

[3] R. Li, Y. Du, Y. Li, Z. He, L. Dai, L. Wang, X. Wu, J. Zhang, J. Yi, *ACS Energy Lett.* **2023**, *8*, 457.

[4] Z. Hao, Y. Zhang, Y. Lu, J. Hou, X. Liu, Z. Yan, J. Chen, *Adv. Funct. Mater.* **2024**, *34*, 2315726.

[5] C. M. Schott, P. M. Schneider, K.-T. Song, H. Yu, R. Götz, F. Haimerl, E. Gubanova, J. Zhou, T. O. Schmidt, Q. Zhang, V. Alexandrov, A. S. Bandarenka, *Chem. Rev.* **2024**, *124*, 12391.

[6] W. Ahn, S. Lee, J. Oh, H. Lee, S.-Y. Choi, *Adv. Mater.* **2025**, *37*, 2413640.

[7] Y. Kang, X. Zhai, Q. Yang, B. Qiao, Z. Bian, H. Chen, H. Hu, Y. Xu, M. Tian, N. Wan, W. Chen, Y. Chai, Y. Zhao, B. Yu, *Innovation* **2025**, *6*, 100885.

[8] C. Teng, Q. Yu, Y. Sun, B. Ding, W. Chen, Z. Zhang, B. Liu, H.-M. Cheng, *InfoMat* **2023**, *5*, e12351.

[9] J. You, Y. Hu, D. Yang, Y. Lin, W. Meng, N. Xu, L. Sun, *Adv. Electron. Mater.* **2025**, *n/a*, 2500221.

[10] M. Prezioso, F. Merrikh-Bayat, B. D. Hoskins, G. C. Adam, K. K. Likharev, D. B. Strukov, *Nature* **2015**, *521*, 61.

[11] Y. Li, Y. Xiong, X. Zhang, L. Yin, Y. Yu, H. Wang, L. Liao, J. He, *Nat. Electron.* **2025**, *8*, 36.

[12] T. Zhang, L. Wang, W. Ding, Y. Zhu, H. Qian, J. Zhou, Y. Chen, J. Li, W. Li, L. Huang, C. Song, M. Yi, W. Huang, *Adv. Mater.* **2023**, *35*, 2302863.

[13] Q. Zhang, Q. Che, D. Wu, Y. Zhao, Y. Chen, F. Xuan, B. Zhang, *Angew. Chem. Int. Ed.* **2024**, *63*, e202413311.

[14] K. Gao, B. Sun, Z. Cao, X. Duan, W. Yan, G. Tong, G. Zhou, X. Chen, J. Shao, *Adv. Funct. Mater.* **2024**, *34*, 2401132.

[15] B. Zhang, W. Chen, J. Zeng, F. Fan, J. Gu, X. Chen, L. Yan, G. Xie, S. Liu, Q. Yan, S. J. Baik, Z.-G. Zhang, W. Chen, J. Hou, M. E. El-Khouly, Z. Zhang, G. Liu, Y. Chen, *Nat. Commun.* **2021**, *12*, 1984.

[16] P. Tang, P. Jing, Z. Luo, K. Liu, X. Zhao, Y. Lao, Q. Yao, C. Zhong, Q. Fu, J. Zhu, Y. Liu, Q. Dou, X. Yan, *National Science Review* **2024**, *11*, nwae322.

[17] G. Xu, H. Cui, L. Wang, M. Zhang, W. Liu, T. Mei, B. Wu, C. Wan, K. Xiao, *ACS Appl. Mater. Interfaces* **2025**, *17*, 34659.

[18] Y. Bu, Z. Ahmed, L. Yobas, *Analyst* **2019**, *144*, 7168.
